# Effects of feeding on different parts of *Ailanthus altissima* on the intestinal microbiota of *Eucryptorrhynchus scrobiculatus* and *Eucryptorrhynchus brandti* (Coleoptera: Curculionidae)

**DOI:** 10.3389/fmicb.2022.899313

**Published:** 2022-08-04

**Authors:** Tian-Chi Ma, Wen-Juan Guo, Jun-Bao Wen

**Affiliations:** Beijing Key Laboratory for Forest Pest Control, Beijing Forestry University, Beijing, China

**Keywords:** gut microbiota, 16S rRNA, weevil, microbe-host relationship, diet niche, microbial diversity

## Abstract

*Eucryptorrhynchus brandti* and *Eucryptorrhynchus scrobiculatus* (Coleoptera: Curculionidae) are two monophagous weevil pests that feed on *Ailanthus altissima* (Mill.) Swingle but differ in their diet niche. In the field, adults of *E. brandti* prefer to feed on the trunk of *A. altissima*, whereas adults of *E. scrobiculatus* prefer to feed on the tender parts. We conducted Illumina sequencing of 16S rRNA to examine changes in bacterial diversity in the adults of these two weevil species after they fed on different parts of *A. altissima* (trunk, 2–3-year-old branches, annual branches, and petioles). Proteobacteria, Tenericutes, and Firmicutes were the dominant phyla in *E. brandti* (relative abundance was 50.64, 41.56, and 5.63%, respectively) and *E. scrobiculatus* (relative abundance was 78.63, 11.91, and 7.41%, respectively). At the genus level, *Spiroplasma*, *endosymbionts2*, *Unclassified Enterobacteriaceae*, and *Lactococcus* were dominant in *E. brandti*, and *Unclassified Enterobacteriaceae*, *Wolbachia* and *Spiroplasma*, and *endosymbionts2* were dominant in *E. scrobiculatus*. Linear discriminant analysis effect size analysis revealed microbial biomarkers in the different treatment group of adults of both weevil species. Adults of *E. brandti* may require the trunk, and adults of *E. scrobiculatus* may require the petioles and annual branches to maintain the high diversity of their gut microbes. The results of this study indicate that feeding on different parts of *A. altissima* affects the composition and function of the microbes of *E. brandti* and the microbial composition of *E. scrobiculatus*. Variation in the abundance of *Wolbachia* and *Spiroplasma* in *E. brandti* and *E. scrobiculatus* is associated with dietary niche changes, and this might explain the evolution of reproductive isolation between these two sibling weevil species.

## Introduction

There is an inseparable relationship between the gut microbes of insects and their hosts, which has been shaped by many generations of natural selection and coevolution (Wilcox et al., 2003). Insects provide habitats for gut microbes, and gut microbes affect their host in various ways. For example, gut microbes can help insects digest food; affect the mating and reproduction of their hosts (Douglas, 2015); provide essential nutrients (Kroiss et al., 2010); improve their resistance to heat and disease (Wilcox et al., 2003), detoxification capacity, and metabolism of foreign bodies (Ramsey et al., 2010); affect the life span and development cycle of their hosts; and promote the development and utilization of resources (Fraune and Bosch, 2010; Kikuchi et al., 2012). For example, the obligatory endosymbiotic bacterium *Buchnera aphidicola* provides essential amino acids and the vitamin riboflavin to the pea aphid *Acyrthosiphon pisum* (Nakabachi and Ishikawa, 1999; Douglas et al., 2011). The tsetse fly *Glossina morsitans* receive nutrients from their obligate endosymbiont *Wigglesworthia glossina* (Nogge, 1981; Akman et al., 2002). Several bacteria have been reported to reduce the vulnerability of their hosts to their natural enemies. For example, *A. pisum* receives protection from the parasitic wasp *Aphidius ervi* when infected with the symbiotic bacterium *Hamiltonella defensa*; they also receive protection from the pathogenic fungus *Pandora neoaphidis* when they possess the bacterium *Regiella* (Oliver et al., 2003, 2005; Scarborough et al., 2005).

The study of insect-gut microbe associations has been a major focus of research; however, few studies have examined weevil-like gut microbes (Blackman and Eastop, 2000; Lyal and Alonso-Zarazaga, 2006; Morera-Margarit et al., 2019). In fact, as the taxon own the most species, weevils possess many endosymbionts and some of it has specialized symbiotic organs, such as *Curculio* and Candidatus *Curculioniphilus buchneri*, *Sitophilus*, and *Sodalis*-allied symbiont (Toju et al., 2010, 2013).

Weevil is a boring pest, which is difficult to control compared with other pests. General management of weevil includes application of chemical pesticides and biological pesticides, e.g., phoxim, pyrethroids, and the spore of *Bacillus thuringiensis* and *Beauveria bassiana* (Aghaee and Godfrey, 2015; Khun et al., 2020). Because of the high cost of insecticides to burrow the bothersome pests and the growing concern about environmental pollution, new environmentally friendly weevil management methods have been sought. After further research on biological learning of weevils, such as the diffusion and transmission, several targeted field management methods have been developed. For example, for rice water weevil *Lissorhoptrus Oryzophilus*, water management, delayed transplanting, and blacklight trapping were adopted to prevent it; for the tree-of-heaven root weevil *E. scrobiculatus*, a novel trunk trap was adopted to prevent it (Chen et al., 2005; Yang et al., 2019).

Although few bacteria are currently used to control weevil, there have been some successful laboratory experiments. After treatment with antibiotics to eliminate *Nardonella*, the body color of *Euscepes postfasciatus* became lighter, and decreased in size, and even after laying eggs on antibiotic-free potatoes, the phenomenon did not disappear in their offspring (Kuriwada et al., 2010; Anbutsu et al., 2017).

The tree-of-heaven root weevil, *E. scrobiculatus* (Coleoptera: Curculionidae), and the tree-of-heaven trunk weevil, *E. brandti*, are two obligate pests of *Ailanthus altissima* (Mill.) Swingle that differs in their diet niche (Yang and Wen, 2021). *Eucryptorrhynchus scrobiculatus* and *Eucryptorrhynchus brandti* have caused severe damage to farm shelterbelts in Ningxia, where *A. altissima* is the main tree species. Previous studies have shown that the adults of *Eucryptorrhynchus scrobiculatus* mostly feed on the petioles, annual branches, biennial branches, and perennial branches of *A. altissima*, whereas the adults of *E. brandti* mostly feed on the trunk; these differences in diet are the main factor underlying the niche differences between the adults of these two weevil species (Ji et al., 2017). When the virgin adults of both species are provided host and non-host materials or different parts (trunk, 2–3 branches, annual branches, and petioles) of the host as nutritional supplements, the number of eggs laid by the adult females is altered, and some are even unable to lay eggs (Zhang, 2015; Guo et al., 2019).

Previous studies have evaluated the relationship between soil microbes and gut microbiota in these two weevil species, and the results of these studies have shown that the gut microbiota of these two weevil species is similar but significantly different from the soil microbiota (Gao, 2021). Thus, there is a need to study the effect of the environment on these two weevil species. Here, the composition and relative abundance of the midgut and hindgut microbiota of two weevil species fed different parts of *A. altissima* (trunk, 2–3-year-old branches, annual branches, and petioles) were investigated to evaluate the different effects of diet of different part on the composition and abundance of gut microorganisms, and whether microbes, in turn, affect the diet or other niches of both weevil species.

## Materials and methods

### Adults of the two weevil species and collection of feeding materials

In September 2021, a total of 240 *E. brandti* adults and 90 *E. scrobiculatus* adults were collected and one young tree of *A. altissima* approximately 6–7 years old was collected in Lingao village, Qingtongxia City, Ningxia (38°4′21.62″N, 106°6′55.82″E). To ensure the freshness of the feed, the trunk of *A. altissima* was placed in a refrigerator at −4°C for storage and replaced every 4 days. The other three parts (petioles, annual branches, and 2–3-year-old branches) were also obtained from the young tree for the first time. The replaced materials were obtained from Shuofang Road, Lingwu City (38°6′ 49.64″N, 106°19 ‘6.04″E) every 2 days (as these parts were more likely to rot in the preliminary experiment).

### Feeding and dissection

All *E. brandti* and *E. scrobiculatus* adults were divided into four treatment groups and a control group. The control group of *E. brandti* and *E. scrobiculatus* was dissected immediately after individuals were collected. In contrast, adults of each treatment group were kept in a plastic box with adequate holes for 15 days. They were fed with the above four different parts (petioles, annual branches, 2–3-year-old branches, and trunk) of *A. sinensis* before being dissected. Feeding materials of *A. sinensis* were cut into small sections (2–3 cm) when fed to weevil adults. The photoperiod was 14 L/10 D, the temperature was 25 ± 1°C, and the humidity was 60 ± 5% RH.

The two species of weevils were dipped in 70% ethanol for 2 min and then cleaned with phosphate buffer saline (PBS, pH = 7.4) three times. The midgut and hindgut of weevils were extracted under an Olympus 311477 (Tokyo) microscope and placed into a 1.5-ml centrifuge tube. The midgut and hindgut were weighed and added to the PBS buffer to make the w/v = 1: 9 (Axelsson et al., 2017). The centrifuge tubes were quickly frozen in dry ice and saved in an ultralow temperature freezer for DNA extraction and library construction.

After being dissected, samples were marked as follows: EbC, *E. brandti* control group; EbT, *E. brandti* fed the trunk; EbB, *E. brandti* fed 2–3-year-old branches; EbA, *E. brandti* fed annual branches; EbP, *E. brandti* fed petioles; EsC, *E. scrobiculatus* control group; EsT, *E. scrobiculatus* fed the trunk; EsB, *E. scrobiculatus* fed 2–3-year-old branches; EsA, *E. scrobiculatus* fed annual branches; and EsP, *E. scrobiculatus* fed petioles. Each group contained three replicates.

### DNA extraction, 16S rRNA amplification, and sequencing

Total DNA was extracted from the midgut and hindgut of dissected insects using the MN NucleoSpin 96 Soil DNA Isolation Kit (Macherey-Nagel, Germany). After extracting the total DNA, DNA quality was assessed using 1% agarose gel electrophoresis. The hypervariable regions V3 and V4 of the bacterial 16S rRNA were amplified using the primer 338F (5′-ACTCCTACGGGAGGCAGCAG-3′) and primer 806R (5′-GGACTACHVGGGTWTCTAAT-3′) with ABI Applied Biosystems 9902 Veriti Thermal Cycler (Applied Biosystems, America). In order to get the sequence linked to primers with Illumina adapters, PCR was performed in 10-μl reaction mixtures containing 4 μl of 5 × KOD FX Neo buffer, 2 μl of 2 mM dNTPs, 0.3 μl of each primer (10 μM), 0.2 μl of KOD FX Neo Polymerase, 50 ng ± 50% of template DNA, and distilled water. The thermal cycling program used was as follows: denaturation for 5 min at 95°C; 25 cycles of 30 s at 95°C, annealing for 30 s at 50°C, elongation for 40 s at 72°C; and a final extension at 72°C for 10 min. Solexa PCR of the 16S hypervariable regions V3 + V4 was performed in 20-μl reaction mixtures containing 2.5 μl of MPPI-a (2 μM), 2.5 μl of MPPI-b (2 μM), 10 μl of 2 × Q5 High-Fidelity DNA Polymerase, and 5 μl of template DNA. The thermal cycling program was as follows: denaturation for 30 s at 98°C; 10 cycles of 10 s at 95°C, annealing for 30 s at 65°C, elongation for 30 s at 72°C; and a final extension at 72°C for 5 min. PCR products were extracted from a 1.8% agarose gel, the voltage was 120 V, and electrophoresis was run for 40 min. An OMEGA purification column and OMEGA bacterial DNA kit (OMEGA, America) were used for over-column purification. The Monarch genomic DNA purification kit (NEB, England) was used for purification after gel cutting. The established library was first inspected, and the qualified library was paired-end sequenced with Illumina NovaSeq 6000. The results include sequence information of the reads and sequencing quality information.

The raw reads obtained by sequencing were filtered by Trimmomatic v0.33 (Bolger et al., 2014). Cutadapt v1.9.1 was used to identify and remove primer sequences, and clean reads without primer sequences were obtained. Usearch v10 (Edgar, 2013) was used to splice the clean reads of each sample, and excessively long sequences in the data were removed. UCHIME v4.2 was used to identify and remove chimeric sequences and obtain effective reads (Magoc and Salzberg, 2011; Beiko et al., 2018).

### Diversity analysis of gut microbiota

16S rRNA gene Illumina sequencing was performed on BMKCloud.^1^ Clustering analysis was performed for the sequences at a 97% similarity level using Usearch, v.10.0, and 0.005% of all sequences were used as the threshold to filter OTUs. The OTUs obtained were compared with those in the Silva v.132^2^ database, and species annotations were determined using the “classify-sklearn” package in the QIIME2 (Bokulich et al., 2017) with a confidence threshold of 0.7. According to data on the microorganisms provided by NCBI,^3^ MEGAN 5^4^ was used to map species abundance information obtained by sequencing on the phylogenetic tree and evaluate the relationship between phylogenetic distances and abundance differences. Alpha diversity indexes were calculated using Mothur v.1.30.2 (Schloss et al., 2009). Student’s *t*-tests were used to detect differences in the indexes between *E. brandti* and *E. scrobiculatus*. The rarefaction curve and bar graphs were generated using the “vegan” package (Oksanen et al., 2010) in R. Beta diversity was estimated using QIIME v.1.9.1 (Caporaso et al., 2010) and visualized using principal coordinate analysis (PCA). The results were plotted using R. Linear discriminant analysis Effect Size (LEfSe) analysis using loose species criteria (species considered different as long as they differed between any two groups) revealed biomarkers (differential microorganism) between groups of *E. brandti* and *E. scrobiculatus*, the Linear Discriminant Analysis (LDA) > 4, and the relative abundance at the classification level of each microflora >0.1%. Nine biomarkers recovered from LEfSe were subjected to a BLAST search to evaluate the relationship between diets and species. LEfSe analysis performed in https://huttenhower.sph.harvard.edu/galaxy/platform used in the non-parametric factorial Kruskal–Wallis (KW) sum-rank test as a first step to detect features with significant differential abundance based on the group of interest in *E. brandti* and *E. scrobiculatus*, the *post hoc* tests were multiple comparisons (all in pairs) using SPSS 26 (Pallant, 2013). The functional contributions of various taxa to different KEGG ortholog groups were determined using the “metagenome contrib” command of PICRUSt2 (Douglas et al., 2018) and visualized in bar plots. The Kruskal–Wallis H-test was used to further confirm microbiological differences between the treatment groups of two weevil species.

## Results

### Sequencing results and OTU clustering

**Figure 1:**
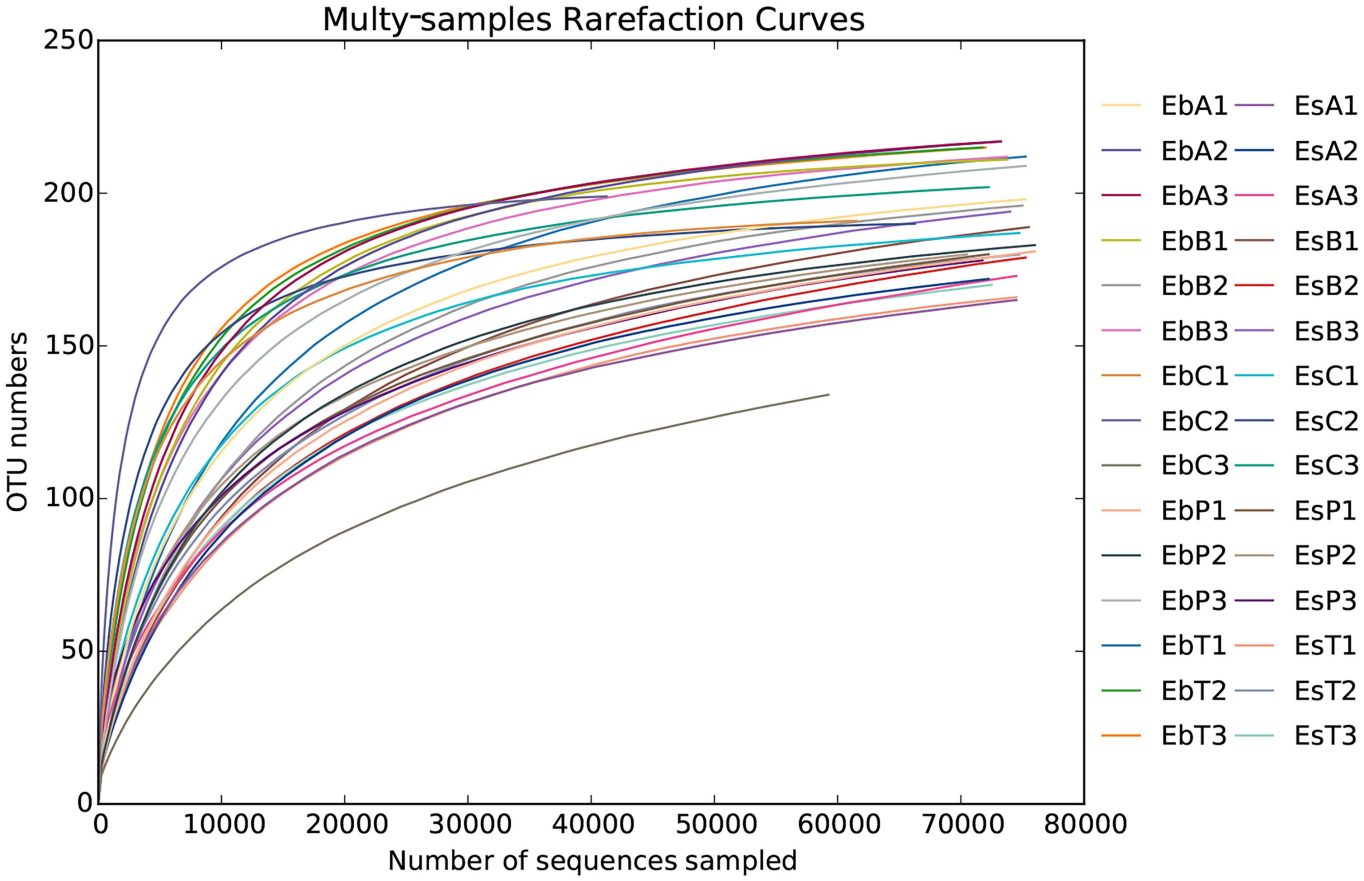
Rarefaction curves of all 30 samples.

**Figure 2:**
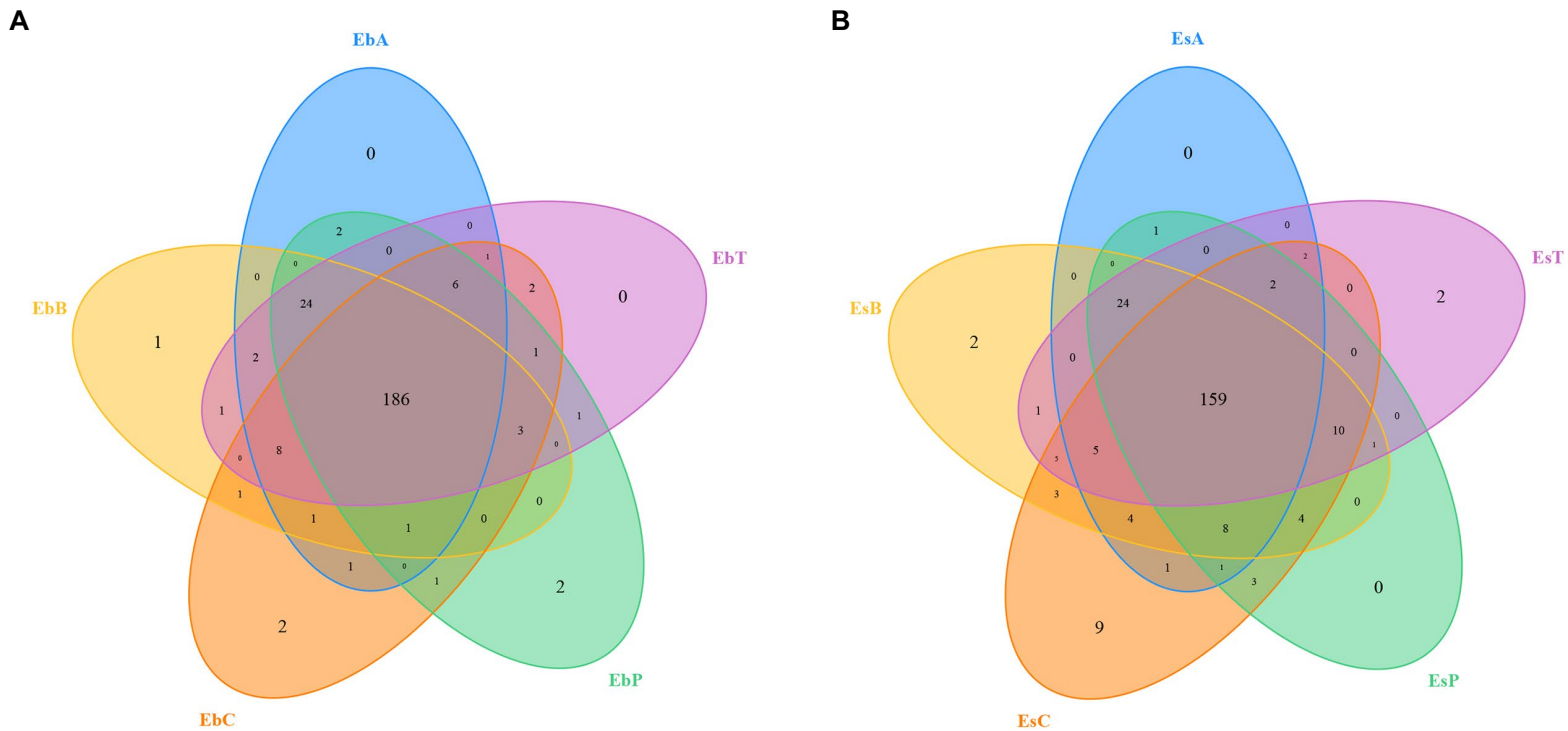
Venn diagram of the OTUs. **(A)** Venn diagram of the OTUs in *Eucryptorrhynchus brandti.* **(B)** Venn diagram of the OTUs in *Eucryptorrhynchus scrobiculatus.*

A total of 2,336,814 pairs of reads were sequenced from 30 samples of the two weevil species, 2,330,938 clean reads were generated after quality control and splicing, and at least 51,587 clean reads were generated from each sample. An average of 77,698 ± 6,370 clean reads was generated. The effective and GC rates of reads are provided in Supplementary Table 1. The sparse curve indicated that the sequencing results were robust and that the sequencing depth was sufficient for most samples (Figure 1). After cluster analysis, a total of 248 OTUs were identified. The number of OTUs from phylum to species is listed in Supplementary Table 2. The number of OTUs in *E. brandti* was greater than that in *E. scrobiculatus*. The number of unique OTUs in the EbC, EbP, and EbB groups was 2, 2, and 1, respectively; there were 9, 2, and 2 unique OTUs in the EsC, EsT, and EsB groups, respectively (Figures 2A,B).

### Alpha and beta diversity

Alpha diversity refers to the diversity within a specific region or ecosystem. The alpha diversity of intestinal microbes in the midgut and hindgut of the two species of weevils was evaluated with the commonly used abundance indexes Chao1, ACE, Simpson, Shannon, and Phylogenetic Diversity (PD; Supplementary Table 3). In *E. brandti*, the Chao1 index was 210.86 ± 14.62; the ACE index was 212.90 ± 15.53, the Simpson index was 0.56 ± 0.10, the Shannon index was 1.94 ± 0.38, and the Phylogenetic Diversity index was 14.25 ± 0.99. While in *E. scrobiculatus*, the Chao1 index was 200.25 ± 10.06; the ACE index was 202.18 ± 12.58, the Simpson index was 0.93 ± 0.87, the Shannon index was 2.54 ± 0.55, and the Phylogenetic Diversity index was 13.30 ± 0.51. The Good’s coverage of all samples was greater than 99.94%, indicating that the sequencing results were sufficient for estimating the diversity of bacterial communities in the two weevil species. There was significant higher species richness in the *E. brandti* that fed on annual branches and the trunk than in the control group according to the Student’s *t*-tests of ACE and Chao1 indexes. In *E. scrobiculatus*, highest Chao1, ACE, and PD indexes and lowest Shannon and Simpson indexes indicated that there was significant highest species richness, but lowest diversities and evenness of microbiota in the groups fed 2–3-year-old branches than other fed groups and control group (Supplementary Figures 1, 2).

**Figure 3:**
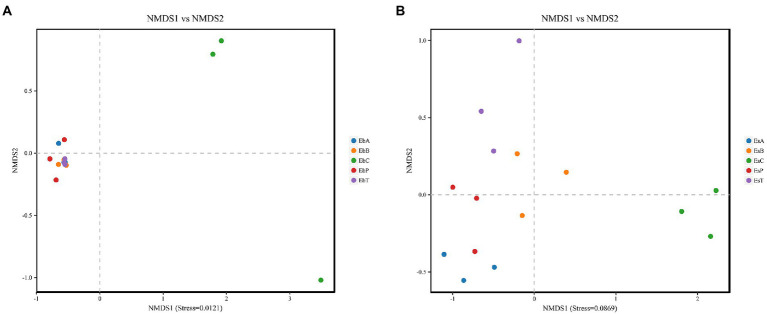
**(A)** Non-metric dimensional scaling (NMDS) analysis in *Eucryptorrhynchus brandti*; **(B)** NMDS analysis in *Eucryptorrhynchus scrobiculatus*. The points in the graph represent different samples, different colors represent different groups, and the distance between points indicates the degree of difference. When the Stress is less than 0.2, the NMDS analysis is considered robust. The proximity of samples on the graph is positively related to their similarity.

Non-metric dimensional scaling (NMDS) was used to determine whether there was a relationship in the diversity between the different groups (Figures 3A,B). Each point on the NMDS graph based on unweighted UniFrac distances represented a different sample. When points of different colors are located in different ranges on the coordinate axis, they are significantly different. Stresses in both weevils were lower than 0.2, indicating that the results were robust enough to distinguish between the different groups. According to NMDS, treatments in *E. brandti* were closely clustered, when the control group is independent. But for *E. scrobiculatus*, the situation was a little different, EsB and EsT were closer and EsP and EsA were closer. The results revealed substantial differences between the control group and the fed groups in *E. brandti* and higher similarity among the fed groups. However, adults of *E. scrobiculatus* fed 2–3-year-old branches and trunk have more similarities in microbial composition, adults fed petioles and annual branches have more similarities.

The unweighted pair-group method with arithmetic means (UPGMA) tree for *E. brandti* and *E. scrobiculatus* based on the unweighted UniFrac method is shown in Supplementary Figure 3. The microbial taxa in the control and fed groups in the two weevil species differed. However, the microbial taxa in the control group were most similar to the microbial taxa in the group fed petioles in *E. brandti*; however, the microbial taxa in the control group were most similar to the microbial taxa in the group fed trunk in *E. scrobiculatus*, this phenomenon seems to suggest that the origin part of their microbial differs from the part of the food that likes to eat.

### Bacterial composition of gut microbiota

**Figure 4:**
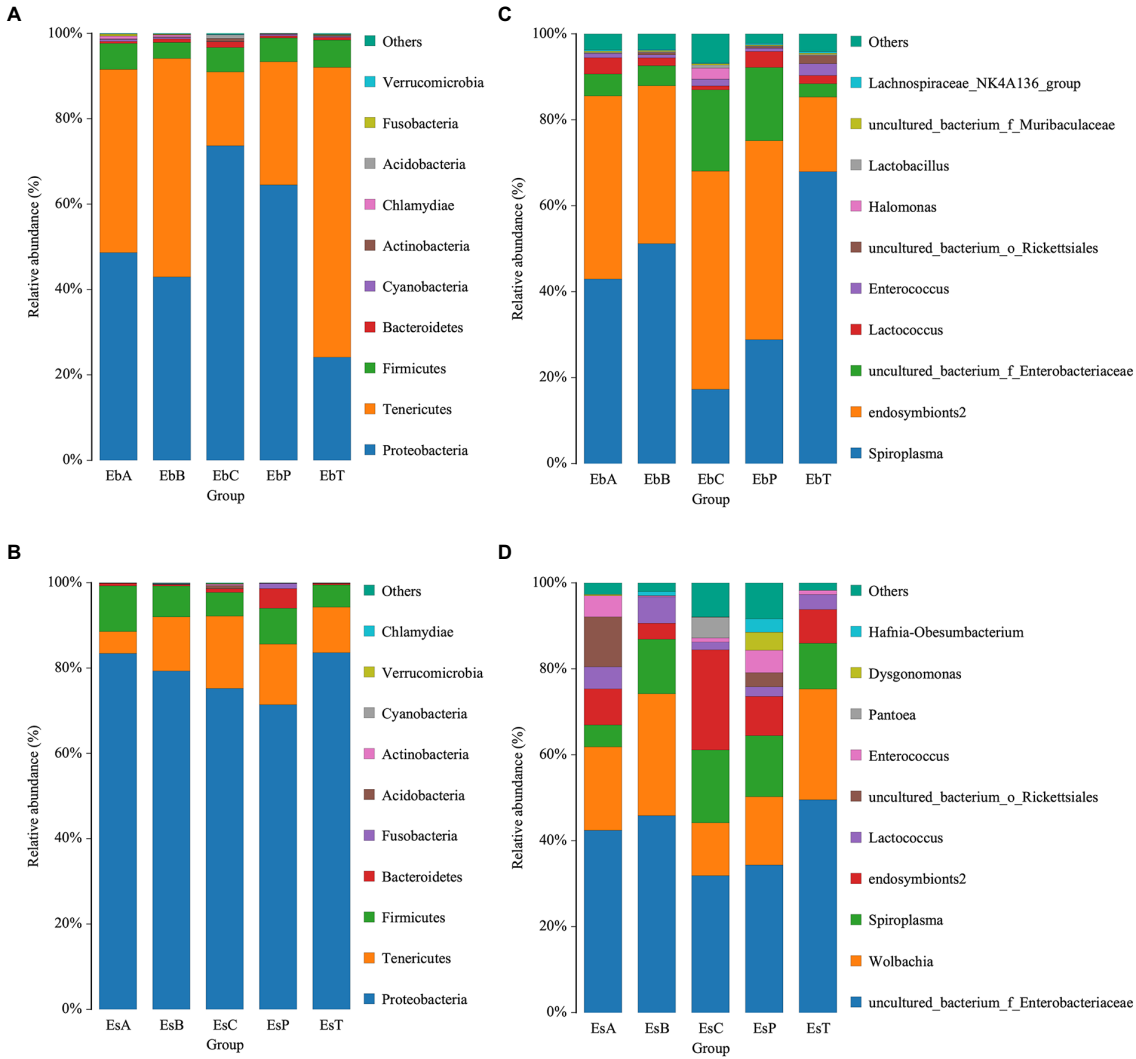
Relative abundance of bacteria communities at the phylum and genus level. **(A)** Relative abundance of bacterial communities at the phylum level in *Eucryptorrhynchus brandti*. **(B)** Relative abundance of bacterial communities at the phylum level in *Eucryptorrhynchus scrobiculatus*. **(C)** Relative abundance of bacterial communities at the genus level in *E. brandti*. **(D)** Relative abundance of bacterial communities at the genus level in *E. scrobiculatus.*

**Figure 5:**
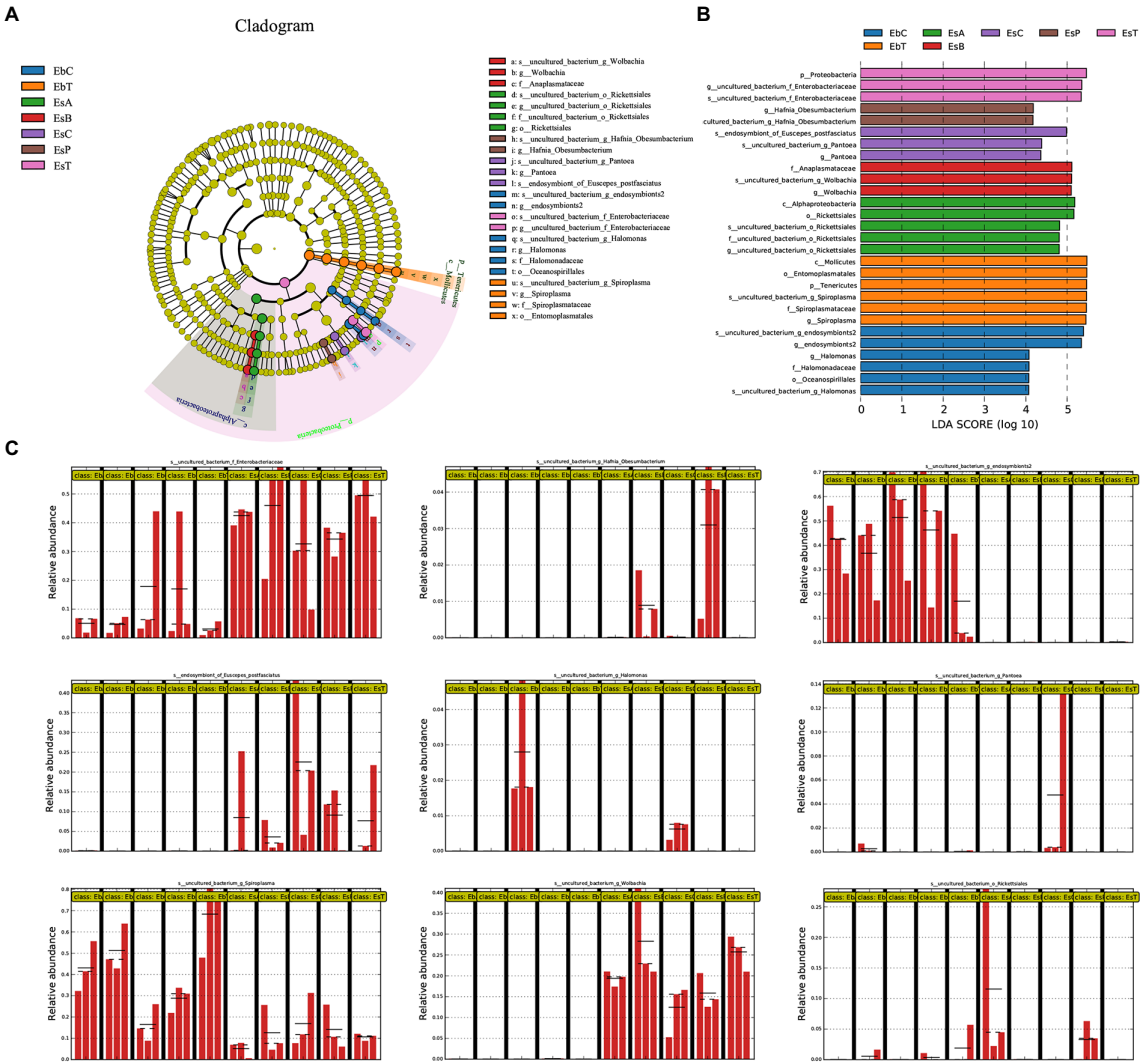
Linear discriminant analysis Effect Size (LEfSe) analysis of samples of *Eucryptorrhynchus brandti* and *Eucryptorrhynchus scrobiculatus*. **(A)** Cladogram of biomarkers in the two species. The circle radiating from the inside out represents the classification level from phylum to species; each small circle at different classification levels represents that level. The diameter of the small circle is proportional to the relative abundance. Species showing no significant differences are in yellow. Biomarkers are colored according to the grouping in which the species is most abundant. **(B)** Linear Discriminant Analysis (LDA) score of biomarkers. **(C)** Relative abundance of nine biomarkers between all groups. Each group from left to right is EbA, EbB, EbC, EbP, EbT, EsA, EsB, EsC, EsP, and EsT. The straight lines indicate the mean. The dotted lines indicate the median.

A bar chart was made to visualize the abundances of the top 10 most abundant phyla and genera. Phyla with a relative abundance greater than 1% were Proteobacteria, Tenericutes, and Firmicutes in *E. brandti* (50.64, 41.56, and 5.63%, respectively) and *E. scrobiculatus* (78.63, 11.91, and 7.41%, respectively). However, the abundance of Firmicutes was higher in *E. brandti* than in *E. scrobiculatus* (Figures 4A,B). The top 10 most abundant genera in *E. brandti* were *Spiroplasma*, *endosymbionts2*, *Unclassified Enterobacteriaceae*, *Lactococcus*, *Enterococcus*, *Unclassified Rickettsiales*, *Halomonas*, *Lactobacillus*, *Unclassified Muribaculaceae*, and *Lachnospiraceae NK4A136 group*. The top 10 most abundant genera in *E. scrobiculatus* were *Unclassified Enterobacteriaceae*, *Wolbachia*, *Spiroplasma*, *endosymbionts2*, *Lactococcus*, *Unclassified Rickettsiales*, *Enterococcus*, *Pantoea*, *Dysgonomonas*, and *Hafnia-obesumbacterium* (Figures 5C,D). The composition of intestinal microbes differed between the two weevil species when they had the same diet; the relative abundances of intestinal microbes also varied among the different diet groups within each weevil species. A phylogenetic tree of the gut microbiota in the two weevil species is shown in Supplementary Figures 4, 5. In *E. brandti*, compared with the control group, *Chlamydiae* was newly distributed in OTU after fed petioles, annual branches, and 2–3-year-old branches; *Roseburia* and *Gemmobacter* were newly distributed after fed all parts of tree; *Sebaldella* was newly distributed after fed 2–3-year-old branches; and *Providencia* was newly distributed after fed petioles and annual branches. In *E. scrobiculatus*, compared with the control group; *Marinifilaceae* was newly distributed in OTU after fed all parts of tree; *Sebaldella* was newly distributed after fed petioles and trunk; *Morganella* was newly distributed after fed petioles and annual branches; and *Orbus* was newly distributed after fed petioles and trunk (Supplementary Figures 4, 5).

### Elucidation of microbial biomarkers

The microorganisms in Figure 5B represent biomarkers, which show significant differences in abundances in one group compared with other microbes are marked in colors. Higher linear discriminant analysis scores indicate higher relative abundance. Figure 5A shows the circle cladogram of biomarkers in the two weevil species. The relative abundance of the nine biomarkers in the different groups is shown in Figure 5C.

In *E. brandti*, the biomarkers at the genus level in the control group (EbC) included *Unclassified Halomonas* sp. and *Unclassified endosymbionts2* sp.; in EbT were *Unclassified Spiroplasma* sp. In *E. scrobiculatus*, the biomarkers in EsC were *Unclassified Pantoea* sp. and *endosymbiont of E. postfasciatus*, while in EsA were *Unclassified Rickettsiales*. Biomarker in EsB was *Unclassified Wolbachia* sp., in EsP was *Unclassified Hafnia-Obesumbacterium* sp., and in EsT was *Unclassified Enterobacteriaceae* sp. An NCBI BLAST search of all the species is shown in Supplementary Table 4. Among the nine different species, BLAST results with similarities greater than 99% were recovered for six species, indicating that the species corresponding to these OTUs were determined. Only the top two most abundant OTUs in *Unclassified Enterobacteriaceae* sp. have been subjected to a BLAST search because their numbers account for over 99% of all OTUs.

The relative abundances of *Spiroplasma* in the five groups of *E. brandti* (Kruskal–Wallis *H*-test, *p* = 0.033) and in *Wolbachia* in the five groups of *E. scrobiculatus* (Kruskal–Wallis *H*-test, *p* = 0.021) were significant. The multiple paired comparison test indicates that *Spiroplasma* is much less abundant in EbC than in EbB (*p* = 0.022) and EbT (*p* = 0.003). The abundance of *Spiroplasma* was lower in EbP than in EbT (*p* = 0.022). The abundance of *Wolbachia* was significantly lower in EsC than in EsB (*p* = 0.014) and EsT (*p* = 0.018); the abundance of *Wolbachia* was lower in EsP than in EsB (*p* = 0.028) and EsT (*p* = 0.036).

### Functional prediction of gut microbiota composition

**Figure 6:**
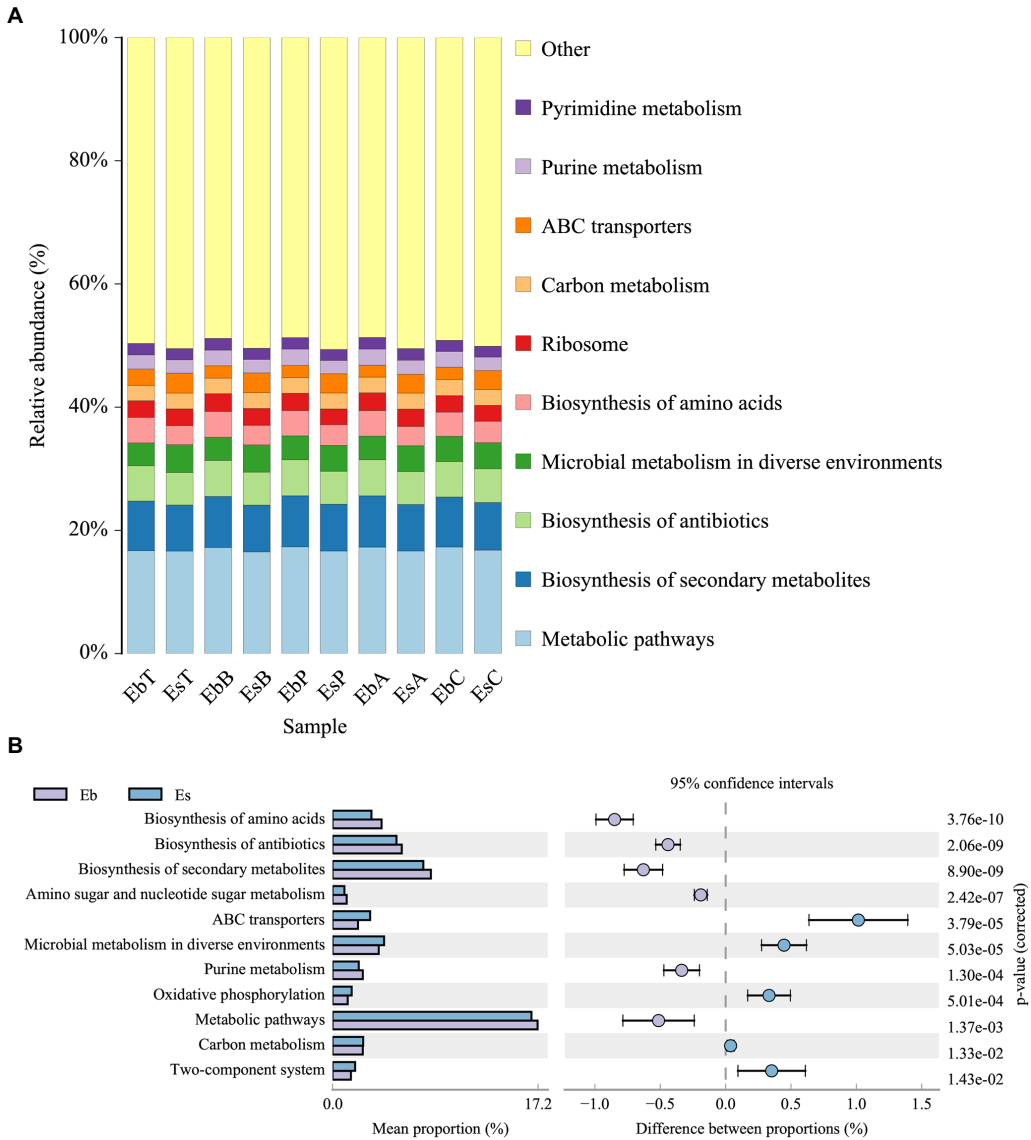
KEGG level 3 function prediction of *Eucryptorrhynchus brandti* and *Eucryptorrhynchus scrobiculatus*. **(A)** Functional composition analysis of *E. brandti* and *E. scrobiculatus.* The top 10 most enriched functions in both weevil species were noted. **(B)** Differential composition analysis of *E. brandti* and *E. scrobiculatus.* Pathways showing relative abundance >1% with 95% confidence in both weevil species are shown.

Based on KEGG level 3 function prediction, the top 10 most enriched pathways in the gut microbiota did not significantly differ between *E. brandti* and *E. scrobiculatus* and were metabolic pathways, biosynthesis of secondary metabolites, biosynthesis of antibiotics, microbial metabolism in diverse environments, biosynthesis of amino acids, ribosome, carbon metabolism, ABC transporter, purine metabolism, and pyrimidine metabolism (Figure 6A). Although variation in the composition of metabolic processes was noticeable in these two species, there were no significant differences between the fed groups and the control group in *E. scrobiculatus*; however, there were significant differences between the fed groups and the control group in microbial metabolism in diverse environments (Kruskal–Wallis *H*-test, *p* = 0.020) and carbon metabolism (Kruskal–Wallis *H*-test, *p* = 0.029) in *E. brandti*. Microbial metabolism in diverse environments was significantly less enriched in EbC than in EbB (*p* = 0.014) and EbT (*p* = 0.003) and in EbP compared with EbT (*p* = 0.036). Carbon metabolism was significantly less enriched in EbC than in EbB (*p* = 0.014) and EbT (*p* = 0.005) and in EbP compared with EbT (*p* = 0.045).

The enrichment of additional 11 pathways (KEGG level 3 functions) differed between *E. brandti* and *E. scrobiculatus* (Figure 6B). The enrichment of the pathways ABC transporters (*p* = 3.79e−5), microbial metabolism in diverse environments (*p* = 5.03e−5), oxidative phosphorylation (*p* = 5.01e−4), carbon metabolism (*p* = 1.33e−2), and two-component system (*p* = 1.43e−2) was greater in the gut of *E. scrobiculatus* than in the gut of *E. brandti*. The enrichment of the pathways biosynthesis of amino acids (*p* = 3.76e−10), biosynthesis of antibiotics (*p* = 2.06e−9), biosynthesis of secondary metabolism (*p* = 8.90e−9), purine metabolism (*p* = 1.30e−4), and metabolism pathways (*p* = 1.37e−3) was higher in the gut of *E. brandti* than in the gut of *E. scrobiculatus*.

## Discussion

The differences between the intestinal microbes of larvae and adults of *E. scrobiculatus* have been previously described, and this work has mainly focused on the differences between the soil microbes and gut microbiota of these two weevil species (Gao, 2021). In this previous study, Proteobacteria (84.68%) and Firmicutes (14.33%) were the main phyla in the midgut of *E. scrobiculatus*, and Proteobacteria (89.70%), Actinobacteria (2.71%), and Firmicutes (1.67%) were the main phyla in the midgut of *E. brandti*. Tenericutes was not one of the main phyla detected in Gao (2021); however, the abundance of Tenericutes in the midgut and hindgut of the two weevil species was high in our study. *Spiroplasma* occurs in the hindgut of *Eurygaster integriceps*, which is consistent with the results of this study (Amiri et al., 2020). Thus, the part of the insect gut sampled and the sampling site can significantly affect the experimental results.

The biomarker species that were found in *E. scrobiculatus* less than (or exclusive to) *E. brandti* include *Unclassified Spiroplasma* sp.*, Unclassified endosymbionts2* sp., and *Unclassified Halomonas* sp.

*Unclassified Spiroplasma* sp. recovered in the BLAST search assigned *Spiroplasma leptinotarsae* with a similarity of 95.58%*. Unclassified Spiroplasma* sp. did not show pathogenicity to the two weevil species in this study, and *Spiroplasma leptinotarsae* is an obligate symbiont of the Colorado potato beetle, *Leptinotarsa decemlineata* (Hackett et al., 1996). So, *Unclassified Spiroplasma* sp. might be in a symbiotic relationship with these two weevil species and are probably as male-killing (MK) reproductive parasites (Werren and O’Neill, 1997; Montenegro et al., 2006; Ammar et al., 2011; Anbutsu and Fukatsu, 2011). *Spiroplasma* sp. in the beetle *Cotinus nitida* has previously been isolated from soil (Clark et al., 1982). The abundance of *Spiroplasma* was higher in *E. brandti* than in *E. scrobiculatus*, especially in the trunk group.

*Unclassified endosymbionts2* sp. recovered in the BLAST search assigned endosymbiont of *Hylobius transversovittatus* with a similarity of 94.01%. This species belongs to the oldest symbiotic genus of the Curculionidae, *Nardonella*. It has been in a symbiotic relationship with weevils for 125 million years. *Nardonella* plays a role in the epidermal synthesis of weevils through the biosynthesis of tyrosine. After treatment with antibiotics, the body color of *E. postfasciatus* became lighter, and decreased in size (Kuriwada et al., 2010; Hosokawa et al, 2015; Huang et al., 2016; Anbutsu et al., 2017; Morera-Margarit et al., 2021). Thus, *Nardonella* provides a promising target for the control of these weevil pests. *Unclassified endosymbionts2* sp. was the most abundant in the control group of *E. brandti*.

*Unclassified Halomonas* sp. recovered in the BLAST search assigned *Halomonas desiderata* with a similarity of 99.77%. *Halomonas* is a salt alkali-resistant bacterium that was exclusively detected in the *E. brandti* and *E. scrobiculatus* controls, but not in any of the treatment groups. This might stem from differences in the laboratory environment and the field (freshness of branches, temperature, and humidity conditions) or differences in the source of the *A. altissima* feeding material. *Halomonas* was probably derived from plant tissue and colonized insect guts (Su et al., 2010). *Unclassified Halomonas* sp. was only observed in *E. scrobiculatus* fed 2–3-year-old branches and petioles.

The microbes that were found in *E. brandti* were less than (or exclusive to) *E. scrobiculatus* include *Unclassified Wolbachia* sp., *Unclassified Enterobacteriaceae*, *Endosymbiont of E. postfasciatus*, *Unclassified Pantoea* sp., *Unclassified Rickettsiales*, and *Unclassified Hafnia-Obesumbacterium* sp.

A BLAST search of *Unclassified Wolbachia* sp. showed that the species was 100% similar to *Wolbachia endosymbiont of Curculio okumai*. *Wolbachia* of *Curculio* spp. is thought to cause cytoplasmic incompatibility (CI; Engelstadter and Telschow, 2009; Merville et al., 2013). A previous study has documented the presence of *Wolbachia* sp. (CI-inducing) in *E. brandti* and *E. scrobiculatus*, and only the *WSP* gene has been investigated to date (Gao et al., 2015). Whether the *Wolbachia* recovered in our study is identical to that identified in this Gao’s study remains unclear. *Wolbachia* was highly abundant in *E. scrobiculatus* but rare in *E. brandti* despite they were collected from the same site. For this result, we propose such a conjecture, *Spiroplasma* and *Wolbachia* in this study may have a competitive relationship. Cause male-killing *Spiroplasma* infection obstructs the spread of CI-inducing *Wolbachia* infection (Yoshida et al., 2019). The ripeness of the feeding material is positively related to the relative abundance of *Spiroplasma* in *E. brandti* and *Wolbachia* in *E. scrobiculatus*. A trunk diet might result in reproductive isolation between *E. brandti* and *E. scrobiculatus*, as the relative abundance of *Wolbachia* and gene flow toward the MK-infected island is increased when the relative abundance of *Spiroplasma* is high (Engelstadter et al., 2008; Yoshida et al., 2019). However, some previous studies have shown that the abundance of *Wolbachia* in the gut is higher in laboratory environments, but the reason for this pattern remains unclear (Liu et al., 2021).

*Unclassified Enterobacteriaceae* recovered in the BLAST search assigned mainly to two related species, *Klebsiella quasipneumoniae*, and *Klebsiella aerogenes*. Previous studies of *K. quasipneumoniae* and *K. aerogenes* in the gut have mainly focused on their metabolic pathways, such as the 2, 3-butanediol pathway in *K. aerogenes*. *K. quasipneumoniae* is resistant to some antibiotics, which facilitates host niche expansion (Azakami et al., 1995; Pagès et al., 2003). Dehydroepiandrosterone, quinone, α-pinene oxide, and carvone are negatively associated with *Klebsiella* in *Monochamus saltuarius*, whereas gentisic acid, mevalonic acid-5P, hydroquinone, and gallic acid are positively associated with *Klebsiella* in *M. saltuarius* (Ge et al., 2021). *Klebsiella* spp. have been found to possess plant-borne proteins (Martínez-Romero et al., 2018). This is consistent with our results that the *Unclassified Enterobacteriaceae* relative abundance of the *E. scrobiculatus* decreases from feeding on the trunk to the petiole.

*Endosymbiont of E. postfasciatus* was the only annotated species among the main intestinal microorganisms of the two weevil species. After the BLAST search, the similarity was 93.97%. This species also belongs to the genus *Nardonella*. *Endosymbiont of E. postfasciatus* was the most abundant in the control group of *E. brandti*.

*Unclassified Pantoea* sp. recovered in the BLAST search assigned to two related species with the same similarity of 99.53%, *Pantoea agglomerans* and *Pantoea vagans* are often isolated from similar locations and co-occur in plant–insect-associated niches. *Pantoea* has been detected in the hindgut of phytophagous insects and is thought to facilitate defense against pathogens through the production of antimicrobial substances. *Pantoea agglomerans* is derived from plants and might be engaged in a facultative symbiosis with grape phylloxera (De Vries et al., 2001; Vorwerk et al., 2007; Palmer et al., 2016). *Pantoea agglomerans* can produce guaiacol in the gut of *Schistocerca gregaria* adults, whereas guaiacol is considered an inhibitor of desert locust aggregation behavior (Dobson et al., 2017; Guo et al., 2020). Aggregation behavior has also been observed in a previous study of *E. brandti* and *E. scrobiculatus* (Ji et al., 2017). The transmission of pathogens from insects to grapes might be blocked at less than detectable levels using antimicrobial peptide-expressing strains of *P. agglomerans* (Arora et al., 2018).

*Unclassified Rickettsiales* recovered in the BLAST search assigned *Hepatincola porcellionum* with the similarity of 88.40%. *Hepatincola porcellionum* was discovered in the midgut gland of terrestrial isopods *Porcellio scaber*, *H. porcellionum* representing a novel lineage among the Rickettsiales (Wang et al., 2004). However, most *Rickettsia* spp. in all arthropods is thought to be pathogenic and soilborne, more information is still needed to confirm the possible role of the microbe (Tsuchida et al., 2010).

*Unclassified Hafnia-Obesumbacterium* sp. recovered in the BLAST search assigned *Hafnia alvei* with the similarity of 100%. Few studies have examined the relationship between *Hafnia* and insects; however, it has previously been isolated from honey (Salimov, 1978). *Hafnia alvei* has been obtained from the honeybee gut and shows antibacterial activity against *Bacillus* sp. It thus may be an opportunistic honeybee pathogen (Tian and Moran, 2016).

Adults of both weevil species in this study tend to move up and down trees, they inevitably come into contact with soil and carry a lot of microbes during the process (Ji et al., 2017). So, the symbiont absence of *Unclassified Halomonas* sp. and *Unclassified Pantoea* sp. in the fed groups likely stems from a lack of contact with the soil. *Unclassified Spiroplasma* sp. is a soilborne symbiont. *Unclassified Hafnia-Obesumbacterium* sp. (*H. alvei*) is also a soilborne bacterium, but it usually causes insect and human disease (Song et al., 2017). *Unclassified Rickettsiales* possibly be a soilborne insect disease microbe. In addition to bacteria, *E. brandti* was reported as a carrier and vector of soilborne fungi that cause plant disease such as verticillium wilt (Snyder et al., 2012).

Although adults of *E. brandti* prefer to feed on the trunk of *A. altissima*, whereas adults of *E. scrobiculatus* prefer to feed on the tender parts. The two weevil species might also need to feed on other parts of *A. altissima* (annual branches for *E. brandti* and 2–3-year-old branches for *E. scrobiculatus*) to maximize the species diversity of their gut microbiota.

The changes in the composition of microbiota might induce changes in metabolic functions (microbial metabolism in diverse environments and carbon metabolism) of *E. brandti*. Carbon metabolism pathway mainly includes carbohydrate metabolism, energy metabolism, and amino acid metabolism. While microbial metabolism in diverse environments mainly includes rumen metabolism, energy metabolism, amino acid metabolism, metabolism of cofactors and nutrition, and xenobiotic biodegradation (mainly aromatics degradation). Previous studies have shown that the carbon-to-nitrogen ratio of the plant trunk is higher than that of the branches (Zhang et al., 2019). *Eucryptorrhynchus brandti* has a stronger carbohydrate and energy metabolism after feeding *A. altissima* than *E. scrobiculatus*, which helps it feed on trunks, while *E. Scrobiculatus* is more suitable for feeding on branches. Aromatics degradation contains catechol cleavage. Changes in microbial function, in turn, might affect the food choices of *E. brandti*, which could eventually result in differences in diet niches.

This study demonstrates that even feeding on different parts of the same host can significantly affect the composition and function of intestinal microorganisms of the same insect species. This is an area that has received little attention in previous studies.

Environmental exposure during early development has been shown to have a significant impact on the gut flora of hosts (Stephens et al., 2016; Dong et al., 2021). Notably, the trunk collection has only one resource and was kept in a refrigerator while other provided plant materials came from two places and were fresher. Thus, the trunk samples used in this study might not provide an accurate representation of their effects.

In sum, we characterized changes in the intestinal bacterial diversity of the adults of two weevil species after they fed on different parts of *A. altissima*. More research is needed to study the role of bacteria in insects. Differentiation of the dietary niche mainly altered the abundance of two reproductive parasites in insects: *Spiroplasma* in *E. brandti* and *Wolbachia* in *E. scrobiculatus*. As a result, the intestinal function of *E. brandti* was altered by feeding on trunks. The difference in the abundances of *Wolbachia* and *Spiroplasma* associated with the diet niche might underlie the reproductive isolation between these two sibling weevil species. In light of the symbiosis with *Wolbachia*, *Spiroplasma*, *Klebsiella*, and *Nardonella* in these two weevil species, potential strategies for controlling the spread of these weevils include applying different *Wolbachia* species and administering antibiotics to remove *Nardonella* from weevil guts (Kuriwada et al., 2010; Gao et al., 2015).

## Data availability statement

The datasets presented in this study can be found in online repositories. The names of the repository/repositories and accession number(s) can be found at: https://www.ncbi.nlm.nih.gov/, PRJNA797527.

## Author contributions

T-CM, W-JG, and J-BW designed the study. T-CM took samples, performed the experiments, and wrote the manuscript. All authors contributed to the article and approved the submitted version.

## Funding

This article was supported by the National Natural Science Foundation of China (Grant No. 32071774).

## Conflict of interest

The authors declare that the research was conducted without any commercial or financial relationships that could be construed as a potential conflict of interest.

## Publisher’s note

All claims expressed in this article are solely those of the authors and do not necessarily represent those of their affiliated organizations, or those of the publisher, the editors and the reviewers. Any product that may be evaluated in this article, or claim that may be made by its manufacturer, is not guaranteed or endorsed by the publisher.

## Footnotes

^1^ https://www.biocloud.net

^2^ https://www.arb-silva.de

^3^ http://www.ncbi.nlm.nih.gov/

^4^ https://ab.inf.uni-tuebingen.de/software/megan5/

## Supplementary material

Supplementary Figure 1 Student’s *t*-test of several indexes in *E. brandti*. **(A)** The ACE indexes. **(B)** The Chao1 indexes. **(C)** Phylogenetic Diversity Whole tree indexes. **(D)** The Shannon indexes. **(E)** The Simpson indexes.

Supplementary Figure 2 Student’s *t*-test of several indexes in *E. scrobiculatus*. **(A)** The ACE indexes. **(B)** The Chao1 indexes. **(C)** Phylogenetic Diversity Whole tree indexes. **(D)** The Shannon indexes. **(E)** The Simpson indexes.

Supplementary Figure 3 UPGMA tree of microbes. **(A)** UPGMA tree of microbes of the control and treatment groups in *E. brandti*. **(B)** UPGMA tree of microbes of the control and treatment groups in *E. scrobiculatus*.

Supplementary Figure 4 Taxa tree of the gut microbiota in *E. brandti*. Each circle of the tree represents a taxon, the size of the circle is scaled logarithmically to represent the number of reads.

Supplementary Figure 5 Taxa tree of the gut microbiota in *E. scrobiculatus*. Each circle of the tree represents a taxon, the size of the circle is scaled logarithmically to represent the number of reads.

Supplementary Table 1 Sequences information of all samples.

Supplementary Table 2 OTUs from phylum to species in all samples.

Supplementary Table 3 Alpha diversity indexes of all samples.

Supplementary Table 4 Blast result of nine different species in ANOVA of LEfSe.
