# Supplementary figures and images for: Effects of feeding on different parts of Ailanthus altissima on the intestinal microbiota of Eucryptorrhynchus scrobiculatus and Eucryptorrhynchus brandti (Coleoptera: Curculionidae)
Source: Front Microbiol. 2022 Aug 4;13:899313. doi: 10.3389/fmicb.2022.899313 (PMC9386226; doi:10.3389/fmicb.2022.899313)

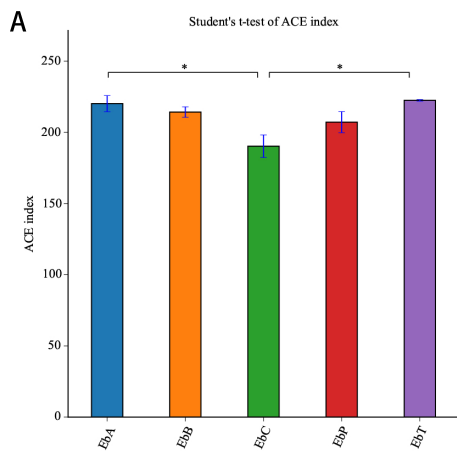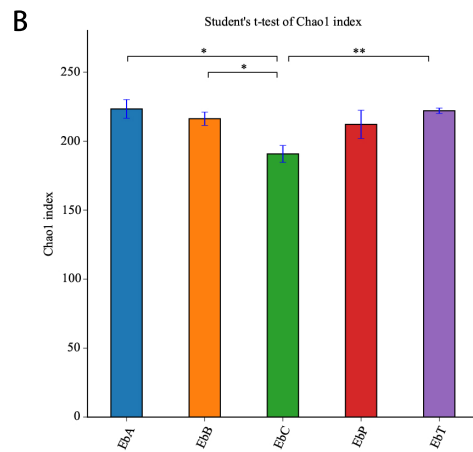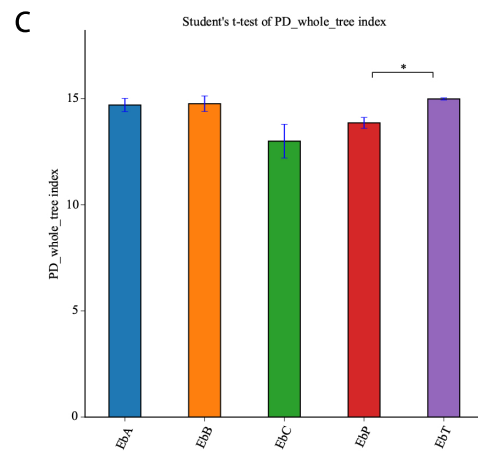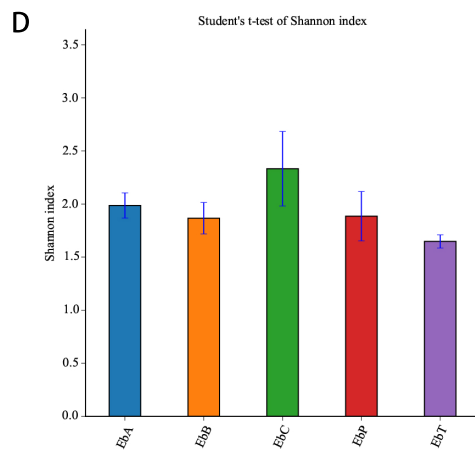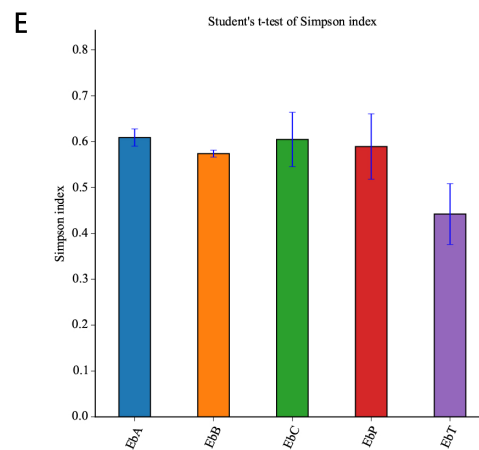

Supplement: Supplementary Figure 1 — Student’s t-test of several indexes in E. brandti. (A) The ACE indexes. (B) The Chao1 indexes. (C) Phylogenetic Diversity Whole tree indexes. (D) The Shannon indexes. (E) The Simpson indexes. [file Data_Sheet_1.PDF]

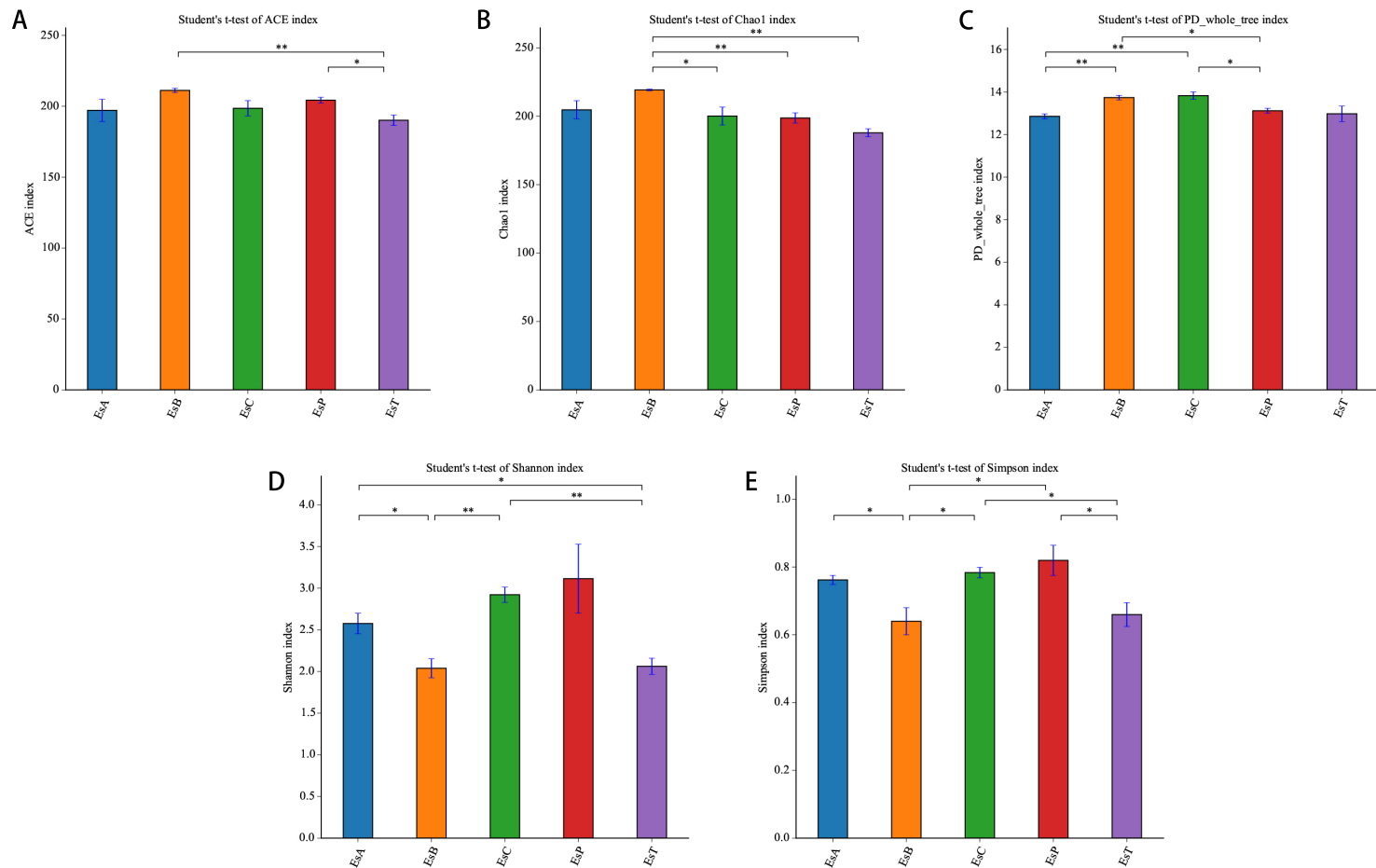

Supplement: Supplementary Figure 2 — Student’s t-test of several indexes in E. scrobiculatus. (A) The ACE indexes. (B) The Chao1 indexes. (C) Phylogenetic Diversity Whole tree indexes. (D) The Shannon indexes. (E) The Simpson indexes. [file Data_Sheet_2.PDF]

A

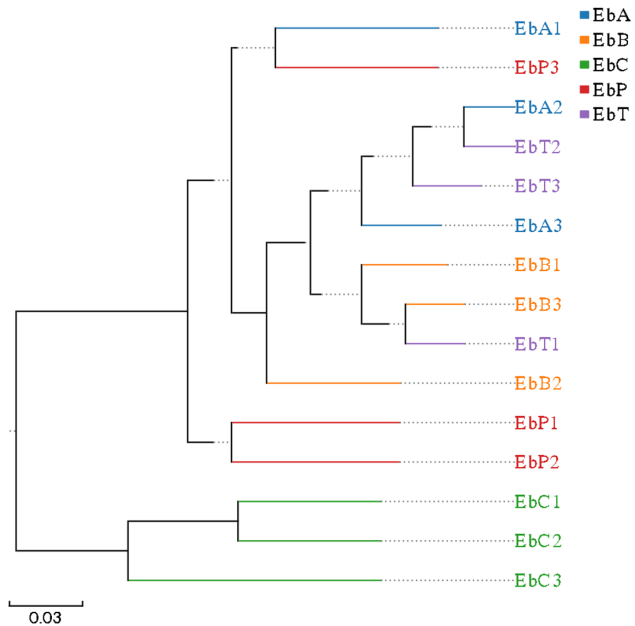

B

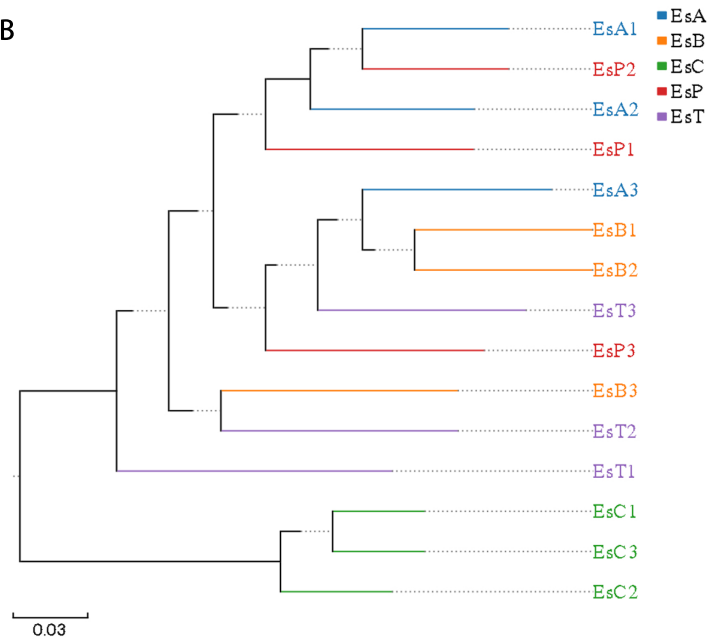

Supplement: Supplementary Figure 3 — UPGMA tree of microbes. (A) UPGMA tree of microbes of the control and treatment groups in E. brandti. (B) UPGMA tree of microbes of the control and treatment groups in E. scrobiculatus. [file Data_Sheet_4.PDF]

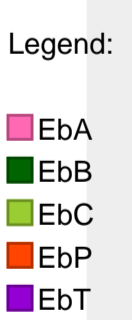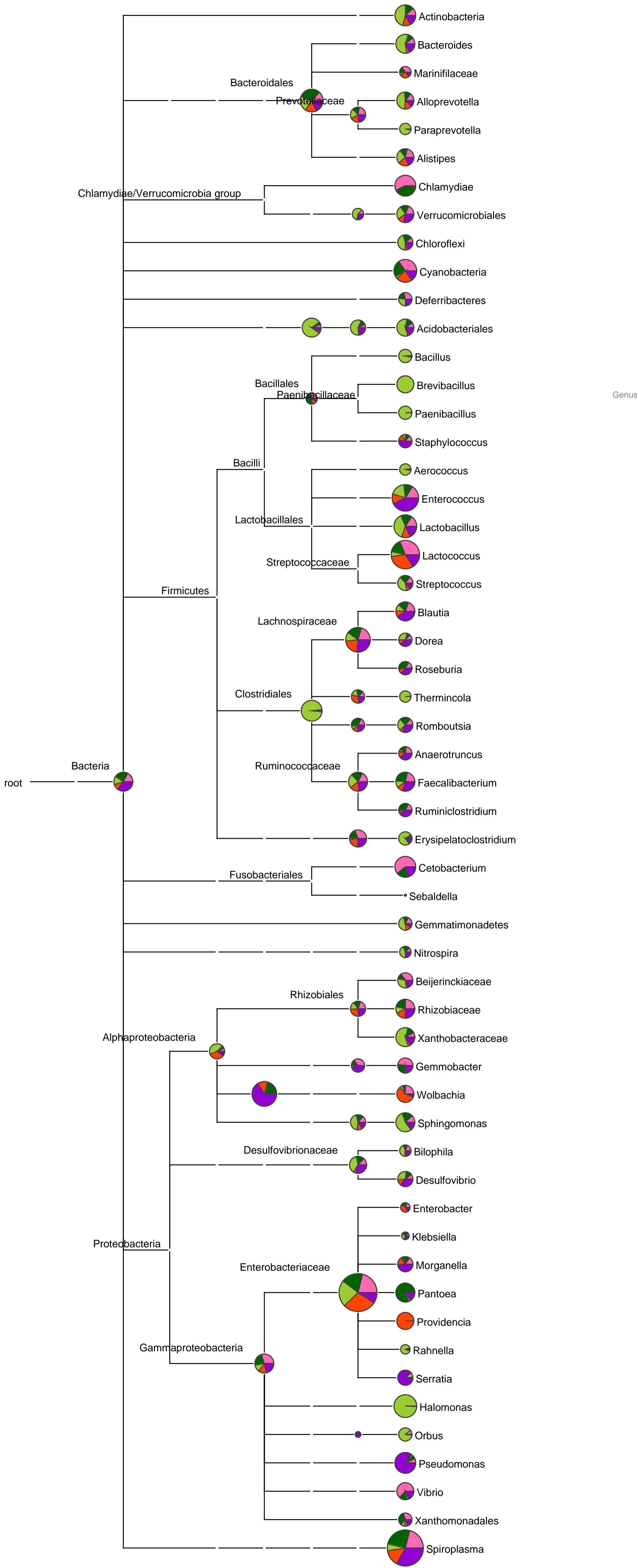

Supplement: Supplementary Figure 4 — Taxa tree of the gut microbiota in E. brandti. Each circle of the tree represents a taxon, the size of the circle is scaled logarithmically to represent the number of reads. [file Data_Sheet_5.PDF]

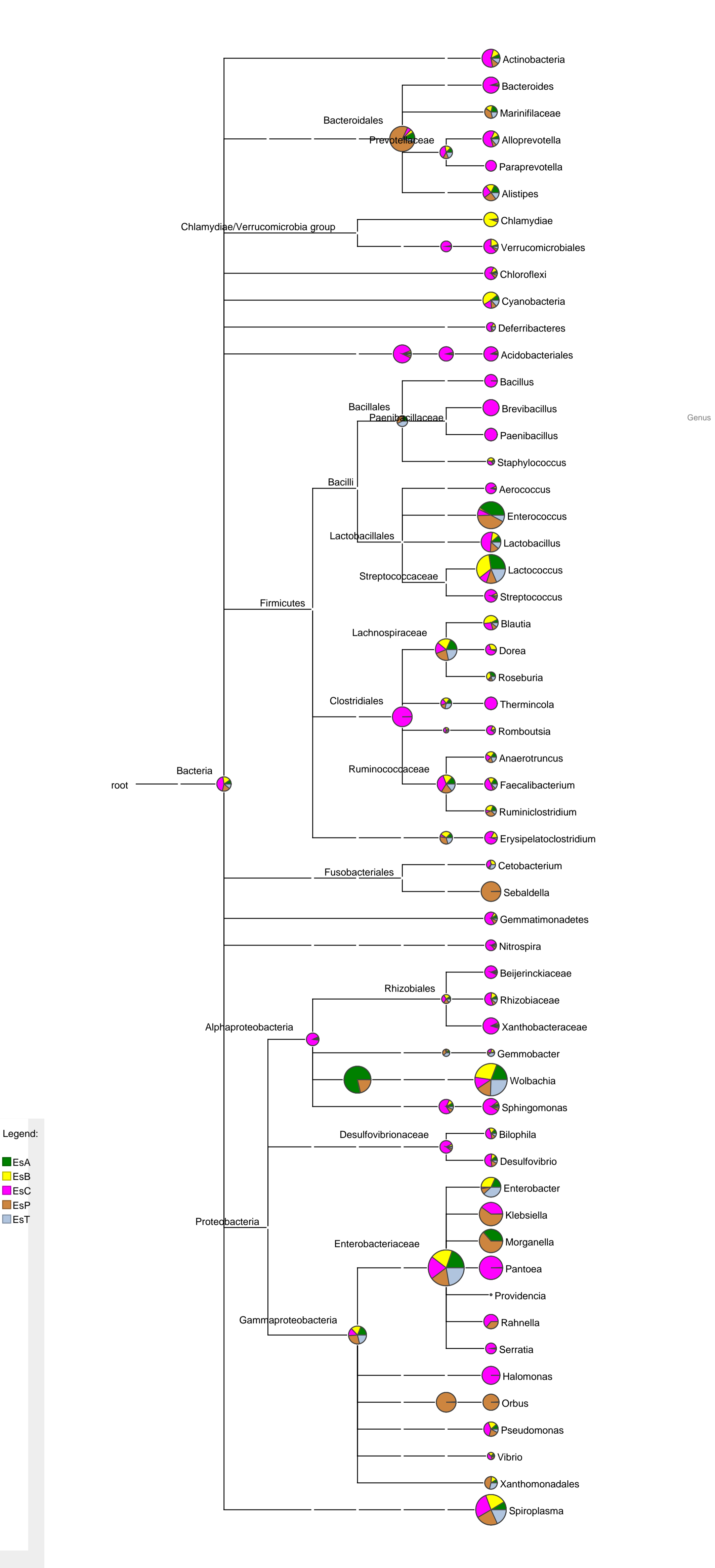

Supplement: Supplementary Figure 5 — Taxa tree of the gut microbiota in E. scrobiculatus. Each circle of the tree represents a taxon, the size of the circle is scaled logarithmically to represent the number of reads. [file Data_Sheet_6.PDF]
