# Supplementary material for: Effects of feeding on different parts of Ailanthus altissima on the intestinal microbiota of Eucryptorrhynchus scrobiculatus and Eucryptorrhynchus brandti (Coleoptera: Curculionidae)
Source: Front Microbiol. 2022 Aug 4;13:899313. doi: 10.3389/fmicb.2022.899313 (PMC9386226; doi:10.3389/fmicb.2022.899313)
Supplement: Supplementary file 10 [file Data_Sheet_3.DOCX]

Cover letter

Dear Editors:

We would like to submit the enclosed manuscript entitled “Effects of Feeding on Different Parts of Ailanthus altissima on the Intestinal Microbiota of Eucryptorrhynchus scrobiculatus and E. brandti (Coleoptera: Curculionidae)”, which we wish to be considered for publication in “Frontiers in microbiology”. No conflict of interest exits in the submission of this manuscript, and manuscript is approved by all authors for publication. I would like to declare on behalf of my co-authors that the work described was original research that has not been published previously, and not under consideration for publication elsewhere, in whole or in part. All the authors listed have approved the manuscript that is enclosed.

The insect gut microbiota is a hot topic. Insects provide habitats for the gut flora, while the latter also affects the host in digest, mate, supplementing essential nutrients, improving resistance etc.

However, the effect of different parts of the same host on the gut microbiota of insects has rarely been studied. This study focused on the changes in intestinal microbial diversity of adults in two weevil species (Eucryptorrhynchus scrobiculatus and E. brandti) with dietary niche differentiation of adults on the same host Ailanthus altissima.

A. altissima is an invasive organism in the United States, and E. brandti has been used for biological control. Understanding these insects gut microbial diversity can help us better control this biological control reagent.

According to the results of species composition and diversity analysis, we found that feeding on different parts of A. altissima affects the composition and function of the microbes of E. brandti and the microbial composition of E. scrobiculatus. Variation in the abundance of Wolbachia and Spiroplasma in E. brandti and E. scrobiculatus is associated with dietary niche changes, and this might explain the evolution of reproductive isolation between these two sibling weevil species.

Our research links different theories from previous research: gut microbes of different insects that feed on the same host may be similar. And feeding on different parts of the plant may eventually cause herbivory insect species differentiation.

Thank you and best regards.

Yours sincerely,

Tianchi Ma

Corresponding author:

Name: Junbao Wen

E-mail: wenjb@bjfu.edu.cn
